# Reduced Survival and Disruption of Female Reproductive Output in Two Copepod Species (*Acartia clausi* and *A. tonsa*) Exposed to the Model Endocrine Disruptor 17α-Ethinylestradiol

**DOI:** 10.3390/toxics11050405

**Published:** 2023-04-24

**Authors:** Tamer Hafez, Fernando Villate, Maren Ortiz-Zarragoitia

**Affiliations:** 1 CBET+ Research Group, Department of Zoology and Cell Biology, Faculty of Science and Technology, Research Centre for Experimental Marine Biology and Biotechnology PiE, University of the Basque Country UPV/EHU, 48620 Plentzia, Basque Country, Spain; 2 MarEsPlank Research Group, Department of Plant Biology and Ecology, Faculty of Science and Technology, Research Centre for Experimental Marine Biology and Biotechnology PiE, University of the Basque Country UPV/EHU, 48620 Plentzia, Basque Country, Spain

**Keywords:** ecotoxicology, endocrine disruptors, *Acartia* species, copepods, zooplankton

## Abstract

Estuaries are heavily impacted by pollutants from different sources such as urban sewage, industrial waste and agricultural runoff. Endocrine-disrupting chemicals (EDCs) are very concerning pollutants to estuarine wildlife, but little is known about their impact on microscopic biota such as zooplankton. The aim of this work was to investigate the effects of a model EDC, the 17α-ethinylestradiol (EE2), on two copepod species inhabiting the Basque coast (Southeastern Bay of Biscay) estuaries: *Acartia clausi* (autochthonous neritic species) and *Acartia tonsa* (non-indigenous brackish species). Female copepods were collected at population maximum time (spring for *A. clausi* and summer for *A. tonsa*) and exposed individually to 5 ng/L (low), 5 µg/L (medium) and 500 µg/L EE2 (high) doses, from environmental concentrations found in sewage effluents to toxicological concentrations. After 24 h exposure, the survival rate of experimental individuals was checked and the lethal concentration LC50 was calculated. The number of egg-producing females and the amount of egg laying and egg hatching were recorded. The integrated biomarker index (IBR) was calculated to integrate the overall effects of EE2 exposure. Both species had reduced survival rates at 500 µg/L, and the LC50 was lower in *A. tonsa* (158 µg/L) compared to *A. clausi* (398 µg/L). The number of eggs laid was significantly reduced in *A. clausi* at EE2 medium and high doses, while a reduction in the number of eggs in *A. tonsa* was observed only at the high dose. However, no significant differences were detected in the egg hatching success of exposed *A. clausi* and *A. tonsa*. IBR index showed that EE2 had the most detrimental effects on *A. tonsa* and *A. clausi* females at the 500 µg/L dose. In conclusion, after 24 h of exposure, EE2 reduced female copepod survival and disrupted reproductive output, but only at high non-environmentally relevant concentrations.

## 1. Introduction

More than fifty percent of the human population inhabits places close to freshwater resources, and more than three billion people live in coastal regions. Rivers and estuaries have been the main source of water supply, but at the same time, they are used for the disposal of sewage and industrial wastes that are finally released into the sea environment, producing severe damage to the ecological status of these water masses and in the organisms inhabiting them [1]. In recent decades, besides the legacy contaminants present in the estuarine and coastal ecosystems, a novel group of emerging contaminants is gaining concern; such are pharmaceuticals and personal care products. These contaminants can act as endocrine-disrupting chemicals (EDCs). EDCs are defined as exogenous substances, which can influence the function of the endocrine system [2]. Such chemicals can mimic or block the actions of certain hormones at their receptors [3]. As a result, EDCs can affect the growth, behavior, reproduction and immune function of a given organism [3].

One of the common EDCs found in estuarine systems in developed countries is the synthetic steroid 17α-ethinylestradiol (EE2), a xenoestrogen analogous to estradiol that has shown high estrogen potency on oral administration. EE2 is used in many formulations of oral contraceptive pills and estrogen therapy and can be released into river, estuarine and marine environments through sewage systems from the urine and feces of patients who consume such medication [4]. In addition, livestock wastes are important sources of EE2 as the steroid can be transferred to water systems through leaching or runoff in rural areas [5]. In the aquatic environment, estrogens are at concentrations of the nanograms per liter level [6]. For example, Ferguson et al. [7] reported that EE2 concentration in a tidal estuarine in southeastern Australia was below 0.20 ng/L. In sediments from a heavily impacted estuarine system in Brazil, EE2 levels were as high as 86.3 ng/g [8]. Despite these low concentrations, estrogenic compounds are biologically active and can affect the reproductive capabilities of several aquatic organisms [9].

Zooplankton communities are essential in estuarine ecosystems as they play a key role in the trophic chain by transferring energy from the primary producers (phytoplankton) towards secondary and tertiary consumers [10]. Due to their importance, any disruption in the zooplankton population can have detrimental effects on the trophic chain. Zooplankton communities have the potential to accumulate contaminants such as EE2 and transfer them to higher trophic levels such as planktivorous fish [11]. Calanoids comprise a relevant group among zooplankton populations in temperate estuarine and coastal waters [12]. They are a key food and energy supply for higher invertebrate and vertebrate (mainly fish) aquatic communities. Calanoids show seasonal dependent fluctuations which affect ecosystem dynamics in estuarine environments, with marked relevance in oligotrophic environments [13,14]. Indeed, they are sensitive to contaminants, and the deleterious effects of selected chemicals on calanoids are known.

The effects of EE2 on several fish species have been well established [15,16]. However, few works have been devoted to analyzing the adverse effects of EE2 on zooplankton species. For example, no significant chronic effects have been observed in the herpactocoid *Nitocra spinipes* after exposure to EE2 for 18 days [17]. In the water flea, *Daphnia magna*, toxicity effects were observed after exposure to EE2 [18]. Such toxicity likely resulted from non-endocrine-mediated responses [18]. In a zooplankton microcosm experiment, exposure to EE2 decreased the species number and thus, the diversity of the zooplankton species [19]. In calanoids, Andersen et al. [20] showed that EE2 had effects on the naupliar development in *Acartia tonsa*. Additionally, Djebbi et al. [21] demonstrated that exposure to EE2 affected the survival, development and sex ratio in *Acartia clausi.*

One of the largest estuarine systems in the north of the Iberian Peninsula, the estuary of Bilbao, was once heavily polluted with metals, organic compounds, PCBs and PAHs due to uncontrolled discharges of untreated mining, domestic and industrial wastes [22]. However, in recent decades, a drastic decrease in the pollution load in the estuary was achieved due to the development of an intensive sanitation process based on sewage collection and treatment and the transformation of the local industry and services [23]. Despite the cleaning efforts, there are still hotspots in the estuary with high concentrations of Pb, Zn, Ni and Cu [22]. In fact, high concentrations of PAHs and PCBs were found in mussel tissues collected from the lower estuary [24]. Near the estuary of Bilbao, the estuary of Plentzia is a smaller estuary with the absence of industrial activity or significant pollution in surrounding areas [25]

For years, *A. clausi* has been the dominant plankton species in the Bilbao estuary [26] and is an important component of the mesoplankton communities in several estuaries of the Basque country as well [14]. However, in recent decades, the invasive species *A. tonsa* became the predominant species in the inner part of the Bilbao estuary [26]. The introduction of *A. tonsa* to the Bilbao estuary was probably through ships’ ballast water exchanges and heavy commercial ship traffic at the Bilbao port. Currently, *A. clausi* is the abundant zooplankton species at the mouth of the Bilbao estuary [27]. Therefore, both *A. clausi* and *A. tonsa* play vital roles in shaping the mesozooplankton communities in the Basque country’s estuarine ecosystem. In addition, any disruption in their population can affect the Basque country’s estuarine ecosystem as a whole.

To our knowledge, no work has compared the sensitivity of these two calanoid species common in the zooplankton of European estuaries, as different species of copepods could have different toxicity profiles towards pollutants such as EE2. Therefore, the aim of this study was to investigate the response of *A. clausi* and *A. tonsa* to a model endocrine disruptor, the 17α-ethinylestradiol (EE2), in terms of the survival and fecundity of exposed females and egg hatching success after exposure.

## 2. Materials and Methods

### 2.1. A. tonsa and A. clausi Collection and Maintenance

Adult females of *A. clausi* and *A. tonsa* used in laboratory experiments were obtained from wild zooplankton samples collected in the outer estuary of Plentzia (43.402717 N, 2.946528 W) and the inner estuary of Bilbao (43.263511 N, 2.924302 W), respectively.

Both *A. tonsa* and *A. clausi* individuals were collected at population maximum periods in May and June 2015. *A. clausi* individuals were collected from the low part of the estuary of Plentzia and *A. tonsa* individuals in July at the upper reaches of the estuary of Bilbao, according to the salinity habitat preferences of each species [28]. *A. clausi* is abundant in high-salinity zones up to 35 practical salinity units (psu), whereas *A. tonsa is* abundant in less saline water of around 30 psu. In both cases, the sampling was conducted two hours after high tide, using a plankton net of 200 µm mesh size. The plankton net was submerged for 10 min under declining tide conditions to filter water masses and retain the zooplankton moving seaward in the direction of the current. *A. clausi* individuals were collected from a 1 m depth in homogenous water column conditions, whereas *A. tonsa* individuals were collected from a 4 m depth, below the halocline, where this species is abundant. After collection, samples were transferred to 10 L tanks containing water obtained at a 1 m depth from the same site where the copepods were collected and immediately transported to the laboratory (in less than one hour) for quick processing. Adult females of *A. clausi* or *A. tonsa* were selected under a stereomicroscope. In the laboratory (Plentzia Marine Station, PiE-UPV/EHU), copepods were transferred to 20 L tanks and were maintained at 18 °C and 33 psu seawater and at a constant photoperiod of a 16/8 h light/dark cycle. In addition, copepods were fed daily with *Tetraselmis chuii* at a concentration of 10,000 cells/mL.

### 2.2. Preparation of 17ɑ-ethinylestradiol Stock and Working Solutions

Three doses of 17α-ethinylestradiol (EE2; Fluka Biochemika co.) were selected for the experimental treatments: 5 ng/L (low), 5 µg/L (medium) and 500 µg/L (high). The low dose corresponds to an environmentally relevant concentration, and medium and high doses have previously been tested in toxicological studies with arthropods and copepods [20,21]. Due to the hydrophobic nature of EE2, acetone was used as a solvent, as proposed by Andersen et al. [20]. EE2 500 µg was dissolved in 100 mL of acetone (stock solution). The final acetone concentrations used in the experimentation were lower than the effect concentrations reported by Andersen et al. [20] (157 mg/L). The stock solution was kept in a cold room at 4 °C. Sequential dilutions of stock solution in 0.2 µm filtered seawater were prepared to obtain the final experimental concentrations of EE2.

### 2.3. Experiment Setup and Studied Endpoints

Prior to exposure, the collected copepods were maintained in clean seawater for 48 h for acclimatization to the experimental conditions. Then, twenty adult females were individually placed in incubation chambers for an exposure period of 24 h using the following experimental conditions: control (no dose) and EE2 low, medium and high doses. Incubation chambers were constructed similarly to the device proposed by Kleppel et al. [29]. The system was divided into two chambers (spawning chamber and brooding chamber). The upper spawning chamber was a 10 cm-diameter plastic tube, which housed the female individuals. A mesh of 200 µm was attached at the base of the tube to prevent female individuals from entering the brooding chamber. The brooding chamber was a 12.5 cm diameter Petri dish attached to the bottom of the spawning chamber to collect the eggs and to house the newly hatched individuals until the end of the experiment. Incubation chambers were attached in glass cups to keep the system in an upright position.

Water containing the corresponding EE2 dose was added to the incubation chambers (50 mL per chamber), and the glass cups were filled with seawater to regulate the temperature in the incubation chamber and maintain the suspension of the experimental system. After the 24 h exposure period, the mortality of exposed females was checked by observing the copepod movement. LC50 value for each species was calculated. To prevent any loss of eggs, which may have not entered the brooding chamber, water was carefully removed from the spawning chamber using a Pasteur pipette with a 45µm mesh attached to the tip. Then, the spawning chamber was carefully separated from the brooding chamber, and the Petri dish was observed under a stereomicroscope. The eggs were counted under the microscope, and the number of eggs laid per female was calculated. The eggs were monitored for hatching every day for three consecutive days. Unhatched eggs beyond the 3-day period were considered unviable. The copepods were fed during the exposure period with *Tetraselmis chuii* at a concentration of 10,000 cells/mL. Experiments were replicated 3 times for *A. clausi* and *A. tonsa*.

### 2.4. Integrated Biological Response (IBR)/n Index

Mortality, the percentage of reproductive females, the average of eggs laid and the egg hatching success were used to create an integrated biomarker response index (IBR). The IBR constitutes a practical and robust tool to assess responsiveness using multiple levels of biological responses [30]. The calculated values of each biological endpoint were plotted using star plots according to [31]. The IBR index was calculated by summing up the triangular surfaces of the star plots (IBR = ∑A_i_) according to the procedure discussed by Beliaeff and Burgeot [31] and modified by Devin et al. [32], which avoids the biomarkers’ order bias in the IBR calculation. As the IBR index is directly dependent on the number of biomarkers in the data set, the obtained IBR index value was divided by the number of biomarkers used [30,31,32,33].

### 2.5. Statistical Analysis

PROBIT analysis was used to calculate the lethal concentration of EE2 causing mortality in 50% of the experimental individuals (LC50) for *A. clausi* and *A. tonsa*, following the protocol described in Vincent [34]. All replicates were pooled for statistical treatment. Data normality and the homogeneity of variance were tested by a Kolmogorov–Smirnov test and Levene’s test, respectively (IBM SPSS v26). Data that did not follow the normal distribution were analyzed using the non-parametric test of Kruskal–Wallis, followed by Dunn’s post hoc test for multiple comparisons (IBM SPSS v26). Data that did follow normal distribution were analyzed using a one-way ANOVA followed by the Tukey–Kramer post hoc test for multiple comparisons (IBM SPSS v26). The relationship between the number of reproductive females and EE2 concentration was analyzed using Chi-square contingency tables (IBM SPSS v26). Contingency table cell residual scores higher than 1.96 were considered significantly different. The egg hatching success for each female was calculated in percentage (%) as the number of nauplii hatched divided by the total number of eggs produced and multiplied by 100 [29]. Finally, Z-score was used to determine the significant difference in the IBR/n index values among the different exposure concentrations.

## 3. Results

### 3.1. EE2 24 h Survival Rate and LC50 Values for Female A. tonsa and A. clausi

**Figure 1:**
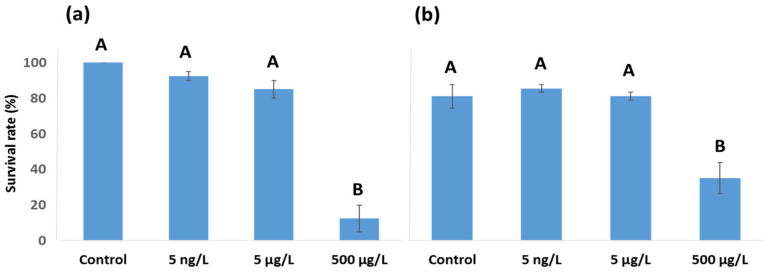
Survival rate of *A. tonsa* (**a**) and *A. clausi* (**b**) after 24 h. Different letters (A or B) denote statistically significant differences (*p* < 0.05) among experimental groups based on one-way ANOVA, followed by Tukey–Kramer post hoc test. Graphs show mean ± standard error (*n* = 20).

Both *A. tonsa* and *A. clausi* showed high survival rates (>80%) at the 5 ng/L and 50 µg/L concentrations (Figure 1a,b). However, in both species, the survival rate significantly decreased at 500 µg/L (one-way ANOVA *p* < 0.05). The survival rates at 500 µg/L were 40% for *Acartia clausi* and 12.5% for *Acartia tonsa.*

Upon calculating 24 h LC50, marked differences were observed in female mortality when comparing both copepod species. According to the probit analysis, the 24 h LC50 for *A. clausi* was 398 µg/L of EE2, whereas the 24 h LC50 for *A. tonsa* was 158 µg/L of EE2.

### 3.2. Effects of EE2 on Reproductive Output in A. tonsa and A. clausi Adult Females

**Figure 2:**
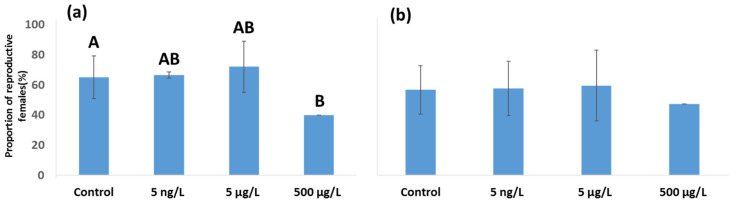
Proportions of reproductive females after 24 h. (**a**) *A. tonsa* females. (**b**) *A. clausi* females. Graphs show mean ± standard deviation. Different letters (A or B) denote statistically significant differences (*p* < 0.05) based on Chi-square adjusted residuals. (*n* = 20).

The proportions of reproductive females in control groups were 65% for *A. tonsa* and 60% for *A. clausi* (Figure 2). No significant differences in the proportion of reproductive females in comparison to controls were detected for the low and medium EE2 doses in both copepod species (Figure 2a,b). However, the high dose (500 µg/L) of EE2 (Figure 2a) was found to have a significant negative effect on the proportion of reproductive *A. tonsa* females (Chi-square *p* < 0.05), which markedly decreased to only 40%, in comparison to control females (Figure 2a). A similar trend was detected for *A. clausi* females exposed to the high EE2 dose, but statistically significant differences could not be observed (Figure 2b).

**Figure 3:**
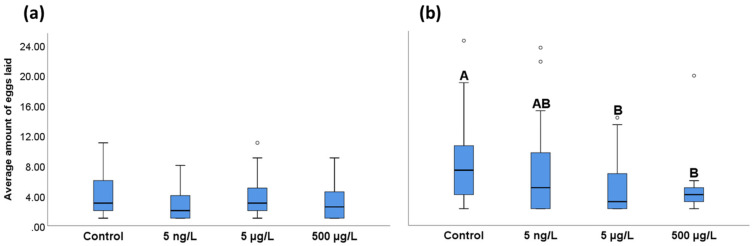
Average number of eggs laid per female. (**a**) *A. tonsa* females. (**b**) *A. clausi* females. Different letters (A or B) denote statistically significant differences (*p* < 0.05) among experimental groups based on the non-parametric test Kruskal–Wallis, followed by Dunn’s post hoc test. Boxes represent the data within the 25th and 75th percentiles; the line indicates the median value; and top and bottom whiskers indicate the minimum and maximum values. Circles indicate outliers. (*n* = 20).

Interspecies variation was observed with regard to the number of eggs laid by *A. tonsa* and *A. clausi* reproductive females (Figure 3). *A. clausi* females laid more eggs per individual than *A. tonsa*, although differences were not statistically significant. *A. tonsa* females exposed to the EE2 conditions did not differ from the control females in egg laying number (Figure 3a). On the other hand, average egg laying per female was significantly reduced after exposure to the medium and high EE2 doses (Figure 3b).

**Figure 4:**
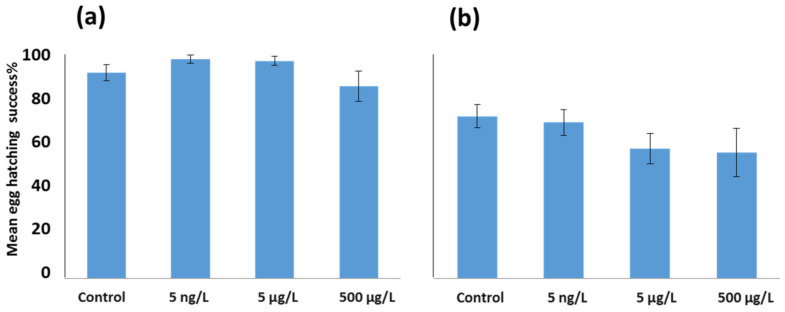
Percentages of egg hatching success in experimental groups. *Acartia tonsa* (**a**) and *Acartia clausi* (**b**). Graphs show mean ± standard error. (*n* = 20).

The percentage of egg hatching success is shown in Figure 4, for both *Acartia* species. *A. tonsa* showed a higher percentage of egg hatching success in comparison to *A. clausi* for all experimental groups. In *A. tonsa* (Figure 4a) the percentage of hatched eggs was similar between the control and the EE2 experimental groups. In *A. clausi*, a trend of reduced egg hatching success was detected after EE2 exposure, although this was not statistically significant (Figure 4b).

### 3.3. IBR/n Index

**Figure 5:**
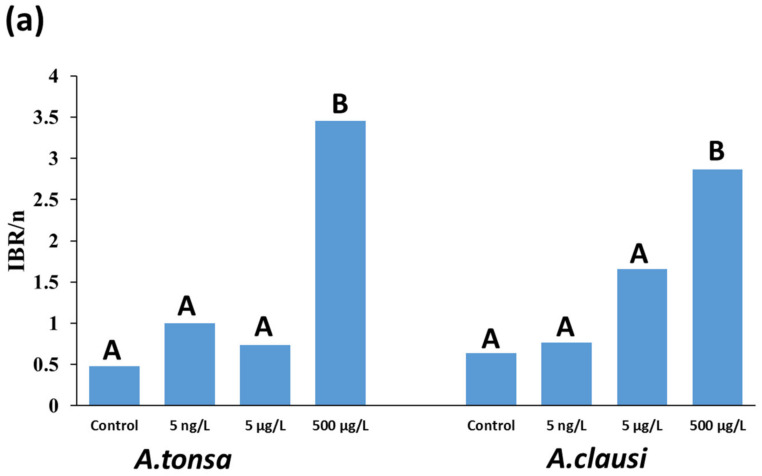

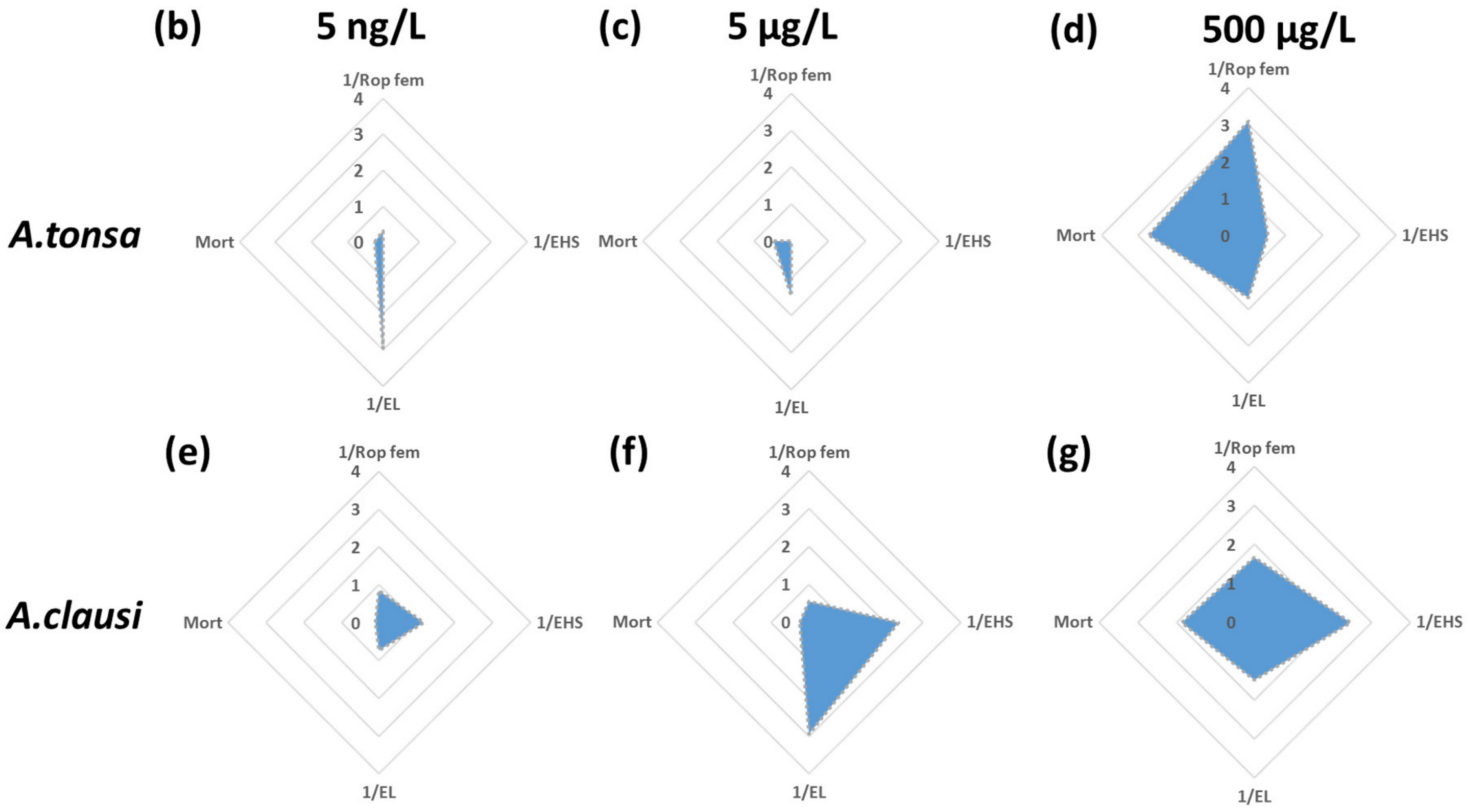
(**a**) IBR/n index calculated based on the surface area highlighted by the dotted lines in the star plots. Different letters denote significant differences between the different treatments according to Z-test (*p* < 0.05). (**b**–**g**) Star plots constructed using the four endpoints calculated in this study for each experimental group of *A. tonsa* and *A. clausi*. The endpoints were survival rate (Mort) reproductive females (Rop fem), egg hatching success (EHS) and the number of eggs produced (EL).

Integrated biomarker response (IBR) values were calculated from the surface area plotted in the star plots (Figure 5b–g). In both *A. tonsa* and *A. clausi*, IBR index values were significantly different at the high dose (500 µg/L) of EE2, highlighting the detrimental effects of the high EE2 dose on the different studied endpoints. In *A. tonsa*, the low number of reproductive females and the high female mortality contributed most towards the high IBR index value. In *A. clausi*, however, the egg hatching success contributed the most towards the high IBR index value.

## 4. Discussion

This study demonstrated that the two congeneric species of calanoid copepods, *A. tonsa* and *A. clausi*, have different sensitivities toward the environmental endocrine disruptor EE2. *A. tonsa* showed higher sensitivity in terms of female survival to EE2 exposure than *A. clausi*, according to survival rates and the 24 h LC50 values. The 24 h LC50 value calculated for *A. clausi* was more than double that calculated for *A. tonsa*. However, female reproductive output was disrupted after EE2 exposure in females from both species. Species-specific responses were observed to EE2 exposure. *A. tonsa* showed a reduced proportion of reproductive females after EE2 exposure, but low or no effect on egg laying proportion or egg hatchability. *A. clausi* showed a decreased egg laying ability and lower egg hatching success after EE2 exposure.

Currently, there are no reported 24 h LC50 values for *A. tonsa* exposed to EE2. Compared to 48 h LC50 in the literature for both species, the 24 h LC50 values obtained from both species in this study were much lower than those previously reported for *A. tonsa,* which has shown a 48 h LC50 of 1.1 mg/L and for *A. clausi* a 48 h LC50 of 1.2 mg/L [20,21]. Different population-specific responses towards natural and anthropogenic contaminants have been reported in copepods [35,36]. This suggests a high sensitivity towards EE2 in the non-indigenous *A. tonsa* and the indigenous *A. clausi* populations in the estuaries of the Basque coast. The interspecies difference in sensitivities toward contaminants depends on several characteristics such as body size, metabolic rate and lipid reserves [37,38]. For example, the average length of *A. clausi* females is 1.13 mm, whereas the average length of *A. tonsa* females is 1.059 mm [39]. The difference in size between the two *Acartia* species can help in explaining the lower 24 h LC50 and the lower percentage of reproduction shown by *A. tonsa* females. The organism’s body length is inversely proportional to the contaminant rate constant, meaning that smaller organisms will show faster responses toward contaminants [37].

Another important factor, which can contribute to observed toxicological differences between the two species, is the history of contamination in the two estuaries. Endocrine disruptors, such as organotin, TBT, phthalate esters, Alkylphenols and 4*t*OP/HHCB musk have been reported in the Bilbao estuary due to urban and industrial discharges [24,40]. In recent decades, however, the pollution loads of the estuary were significantly reduced [41,42]. However, until now, there is no available information on the concentration of EE2 in the estuaries of Bilbao and Plentzia. Therefore, updated information is needed regarding the chemical and physiochemical properties of both estuaries to fully understand the toxicological differences observed between the two species.

The significant decrease in eggs produced by *A. clausi* after exposure to 5 µg/L and 500 µg/L was similar to the results obtained by Djebbi et al. [20] in which the authors reported a significant reduction in the egg production rate after exposure to 100 µg/L EE2. Interestingly, contrary to our observed results with *A. tonsa*, Andersen et al. [43] demonstrated that a dose of 23µg/L of the natural estrogen 17-β estradiol stimulated the growth of the ovaries in *A. tonsa* and had a significant increase in egg production compared to the control. Natural estrogenic steroids can stimulate the maturation of female internal reproductive organs in several crustacean species [43]. However, the synthetic hormone EE2 is a much more potent estrogen agonist than its natural counterpart. EE2 is shown to be twenty times more potent than estradiol in inducing vitellogenic effects in some species of fish [44].

Egg hatching success in general was different between *A. tonsa* and *A. clausi,* being slightly higher in *A. tonsa*. Egg hatching success was previously reported to be significantly reduced after *A. clausi* females were exposed for 48 h to EE2 at 50 ng/L and 5 µg/L doses, but not at such high doses as 50 µg/L and 500 µg/L [20]. The slight reduction in hatching success observed for *A. clausi* in this study after 24 h of exposure might allow further speculation that longer exposure would further reduce egg hatching success in higher doses. For example, *A. tonsa* females exposed for 7 days to marine gas oil water accommodated fraction (WAF) showed a significant reduction in egg hatching success at the highest dose [45]. In addition, the generally high egg hatching success among all treatments might not be a definite indicator that the offspring will develop properly.

As shown from our results, the low and environmentally relevant concentrations will not have any adverse effects on the fecundity of both copepod species after a short exposure period (24 h). The average concentration of EE2 in sewage waters is around 5 ng/L whereas heavily polluted waters can reach concentrations of EE2 up to two or three times such values [46]. On the other hand, the water flea, *Daphnia magna,* exposed to environmentally relevant concentrations of EE2 (10 ng/L and 100 ng/L) for 21 days showed a significant decrease in nauplii production [47]. Additionally, Rodrigues et al. [48] reported significant decreases in somatic growth rates in *Daphnia magna* exposed to EE2 at a concentration of 100 ng/L for 21 days. Compared to *A. tonsa, Daphnia magna* was shown to be up to twofold less sensitive towards contaminants such as oil water accommodated fractions [49]. The sensitivity of *A. tonsa* towards contaminants highlights the possibility of observing toxicity effects at environmentally relevant EE2 concentrations after chronic exposure scenarios. Therefore, chronic and transgenerational studies are crucial in order to fully understand the effects of EE2 on the development and the fecundity of the zooplankton population.

## 5. Conclusions

In conclusion, this work has demonstrated the toxicological effects of 17α-ethinylestradiol (EE2) on two species of copepods common in two Basque estuaries with different pollution loads. High doses of EE2 affected the reproductive endpoints differently in the two species and induced mortality. It is important to mention that low and environmentally relevant concentrations did not have any adverse effects on reproduction capabilities. However, chemical analysis of EE2 concentrations in the two Basque estuaries is crucial to fully understand the effects of EE2. In addition, an extensive transgenerational study on the two copepods is necessary in order to assess the effects of EE2 on the developmental stages of copepods. Still, this study has shed the light on some of the detrimental effects of EE2 on the estuarine environment and consequently, stricter regulations from governments should be implemented on the applications of estrogenic compounds. For example, stricter regulations should be implemented on waste disposal from livestock treated with estrogenic compounds to prevent such compounds from spilling into water systems.

## Data Availability

Data available on request to the corresponding author.
